# Trends in Sodium Content of Instant Foods in Thailand, 2018–2021: Progress and Remaining Challenges

**DOI:** 10.5888/pcd23.250382

**Published:** 2026-07-16

**Authors:** Chanatip Chailek, Patcharanee Pavadhgul, Wirin Kittipichai, Surasak Kantachuvesiri

**Affiliations:** 1 Master of Public Health Program, Faculty of Public Health, Mahidol University, Thailand; 2 Faculty of Public Health, Mahidol University, Thailand; 3 Faculty of Medicine, Ramathibodi Hospital, Mahidol University, Thailand

## Abstract

**Introduction:**

Excess sodium intake is a major risk factor for hypertension and cardiovascular disease. Thailand has implemented sodium-reduction strategies, including the Salt and Sodium Reduction Strategy (2016–2025), front-of-package labeling, and the Healthier Choice logo. This study aimed to describe trends in sodium content of instant foods sold in Thailand from 2018 to 2021.

**Methods:**

We conducted an analytical study using secondary data from 2 packaged-food surveys in retail stores in the Bangkok Metropolitan Region, Thailand, in 2018, 2019, and 2021 administered by the Thai Food and Drug Administration and the Low Salt Network. We applied descriptive statistics, Mann–Whitney U, Kruskal–Wallis, Wilcoxon signed-rank, and χ^2^ tests, with *P* < .05 considered significant.

**Results:**

We analyzed data for 788 products. In 2021, the median (IQR) sodium content was 1,750.0 (1,289.1–2,267.3) mg/100 g, an 11.6% decrease from 2018 (*P* = .02). Overall, we found significant reductions of sodium in rice porridge/soup, brand C, egg noodles, cup products, and items manufactured in Thailand and China. Conversely, among 57 identical products available in both 2018 and 2021, median sodium content did not change significantly (*P* = .06); 33.3% decreased, 19.3% increased, and 47.4% were unchanged. The proportion of Healthier Choice products increased from 4.5% in 2018 to 21.1% in 2021 (*P* < .001), including both noodles and rice porridge/soup.

**Conclusion:**

Sodium levels in instant foods in Thailand declined modestly, and Healthier Choice products increased substantially. However, most items remain above World Health Organization benchmarks, with inconsistent reformulation. Revising Healthier Choice criteria, setting mandatory sodium targets, and strengthening monitoring could accelerate sodium reduction and improve cardiovascular health.

**Summary**

**What is already known on this topic?**

Excess sodium intake is a leading risk factor for hypertension and cardiovascular disease, and instant foods are a major source of dietary sodium in Thailand.

**What is added by this report?**

This study found a modest decline in sodium content of instant foods from 2018 to 2021 and a significant increase in products meeting Healthier Choice criteria, although most items remain above World Health Organization benchmarks.

**What are the implications for public health practice?**

Strengthening sodium targets, revising Healthier Choice thresholds, and systematic monitoring are needed to accelerate sodium reduction in Thailand’s food supply.

## Introduction

Cardiovascular diseases are the leading cause of mortality worldwide, responsible for 32% of global deaths (1). In Thailand, noncommunicable diseases account for most premature deaths, and hypertension is a principal modifiable risk factor (2,3). Excess sodium intake contributes substantially to hypertension and cardiovascular diseases, with the Global Burden of Disease Study estimating 70 million disability-adjusted life years attributable to high sodium diets in 2017 (4). The World Health Organization (WHO) recommends sodium intake below 2,000 mg per day, yet Thai adults consume an average of 3,636 mg of sodium per day (5,6). Although condiments are the primary source of dietary sodium, processed or packaged foods contribute approximately 20% of total intake, with instant noodles being the most frequently consumed item in this category (59.7%) (7).

Thailand consistently ranks ninth globally in instant noodle consumption, reaching 3.7 billion servings in 2020. Despite minor market fluctuations during the COVID-19 pandemic, absolute consumption remained exceptionally high, growing to 4.0 billion servings by 2024 (8). Surveys during 2012 through 2015 showed sodium levels ranging from 1,000 to 2,000 mg per serving, among the highest for packaged foods (9,10). In response, Thailand introduced the Salt and Sodium Reduction Strategy (2016–2025) in 2016 (9), along with the Healthier Choice logo (11) and mandatory guideline daily amount (GDA) labeling for instant foods (12). The Healthier Choice logo is a voluntary front-of-package nutrition symbol administered by the Institute of Nutrition, Mahidol University, to certify products meeting category-specific nutrient criteria and help consumers identify healthier options (13). In 2018, the Thai Food and Drug Administration (FDA) revised the Thai recommended daily intake for sodium from 2,400 to 2,000 mg per day (14), and the Thailand Sodium Reduction Initiative set voluntary reformulation targets with the food industry (15).

Despite these strategies, the sodium content of instant foods has not been systematically evaluated since these policies were implemented. Monitoring these products is critical for evaluating national progress toward WHO’s global target of a 30% reduction in population sodium intake. The main objective of this study was to describe trends in sodium content of instant foods sold in Thailand from 2018 to 2021. Secondary objectives were to examine changes in sodium content of identical products and the proportion of products meeting Healthier Choice criteria.

## Methods

### Study design

We conducted an analytical study using secondary data from 2 serial cross-sectional packaged-food surveys in retail stores in the Bangkok Metropolitan Region, Thailand, from 2018 to 2021.

### Data sources and population

We identified products from 2 sources: 1) the Situation Survey and Development of Guideline Daily Amount Labeling conducted in 2018–2019 by the Division of Food, Thai FDA, and 2) the Monitoring Sodium Content in Ready-to-Eat Meals and Instant Foods for a Sodium Reduction Campaign conducted in 2021 by the Low Salt Network and the Kidney Friends Association of Thailand. To ensure a representative sample of products available to Thai consumers, both surveys used consistent data collection protocols. Data collectors visited purposively selected major retail chains — comprising supermarkets, hypermarkets, and convenience stores — chosen based on their dominant market share in the region.

Eligible products were instant foods — defined as noodles or rice porridge/soup requiring minimal preparation — sold in Thailand with nutrition labels and manufactured domestically or imported. Products were excluded if they were concentrated soups, seasoning cubes, powders, or chili pastes; if duplicates occurred within the same year; or if essential data (net weight, serving size, or sodium content) were missing.

### Data extraction and management

We recorded data in Microsoft Excel. We extracted data on the following variables: survey year, product name, brand, recipe, food serial number, noodle type, container type, preparation method, country of manufacture, net weight, serving size, sodium content, and Healthier Choice status. We used the Thai FDA’s product licensing database and retail websites to fill in missing values for brand, recipe, container, preparation, and country of manufacture. We identified products certified as Healthier Choice by matching survey data with official lists from 2015–2018 and 2019 (16,17) and by the presence of the Healthier Choice logo on product labels during the survey in 2021.

We consolidated data into a single dataset. Duplicate records were removed using serial numbers, incomplete records excluded, and variable definitions standardized. We matched identical products between 2018 and 2021 by using serial numbers as unique identifiers.

### Outcomes and variables

The primary outcome was sodium content, which was standardized to mg per 100 g of product (mg/100 g) to account for differences in product sizes. Secondary outcomes were changes in sodium content of identical products from 2018 to 2021 and the proportion of products meeting Healthier Choice criteria. We classified product characteristics by type (noodles vs rice porridge/soup), recipe (spicy vs nonspicy), container (bag vs cup), preparation (with soup vs without soup), and country of manufacture. We anonymized and categorized brand names as A, B, and C, representing the 3 leading manufacturers with the highest cumulative market share in the Thai instant food market during the study period, while all other brands were grouped as “others.”

### Statistical analysis

We used descriptive statistics to summarize product characteristics (frequency, percentage, mean, median, IQR, and range). We conducted between-group comparisons for nonnormally distributed variables (net weight, serving size, sodium content) with the Mann–Whitney U or Kruskal–Wallis test, followed by the Dunn test for multiple comparisons. To quantify the magnitude of changes between survey years, we calculated and reported effect estimates as the percentage change in median sodium content. A logarithmic regression analysis modeled trends in sodium reduction from 2015 to 2021, with *R* ^2^ values used to assess model fit. We used the median sodium content from a 2015 survey of Thai ready-to-eat packaged foods (10) as a baseline. This was the only such value available before our study, and it preceded Thailand’s main sodium-reduction policies. The Wilcoxon signed-rank test assessed within-product sodium changes from 2018 to 2021. We used χ^2^ or Fisher exact tests to compare proportions of Healthier Choice products. Statistical significance was set at *P* < .05.

### Ethical considerations

This study was approved by the Ethics Review Committee for Human Research, Faculty of Public Health, Mahidol University (protocol no. 25/2565). Data use was authorized by the Thai FDA, Low Salt Network, and Kidney Friends Association. Specific brand names were withheld to maintain the study’s focus on industry-wide reformulation progress rather than individual commercial performance, in alignment with the data-sharing agreements.

## Results

### Characteristics of products

We analyzed 788 instant food products: 201 in 2018, 302 in 2019, and 285 in 2021. Across all years, most products were noodles (83.2%–85.4%); the remainder was rice porridge/soup. Median (IQR) net weight decreased from 104 (60–210) g in 2018 to 74 (55–120) g in 2021 (*P* < .001), largely due to smaller noodle packages. Median (IQR) serving size was stable overall at 60 (46–90) g in 2018 and 60 (47–85) g in 2021, with a slight increase for rice porridge/soup, from 35 (30–35) g to 40 (32–42.5) g (*P* = .03). By type of noodles, egg noodles declined from 56.2% of products in 2018 to 37.5% in 2021 (*P* < .001), while ramen increased from 11.4% to 24.2% (*P* < .001). Products prepared with soup decreased from 82.6% in 2018 to 72.6% in 2021 (*P* = .03).

### Sodium content in 2021

The median (IQR) sodium content in 2021 was 1,750.0 (1,289.1–2,267.3) mg/100 g (Table 1). By type of noodle, glass noodles had the highest sodium content (2,896.1 mg/100 g), while “other” noodles had the lowest (1,225.0 mg/100 g). Products with soup contained more sodium than those without soup (1,913.0 vs 1,076.9 mg/100 g, *P* < .001). By country of manufacture, Japanese products had the highest sodium content (2,909.0 mg/100 g), while South Korean products had the lowest (1,265.2 mg/100 g, *P* < .001).

**Table 1:** Sodium Content (mg/100 g) of Instant Foods Sold in Thailand, 2021

Characteristic	No.	Mean (SD)	Median (IQR)	Range	*P* value^a^
**Total**	285	1,854.0 (1,016.1)	1,750.0 (1,289.1–2,267.3)	0–8,000.0	NA
**Type**					
Noodles	237	1,926.7 (1,016.9)	1,777.8 (1,300.0–2,333.3)	190.0–8,000.0	.08
Rice porridge/soup	48	1,495.2 (942.5)	1,746.4 (866.1–1,954.8)	0–3,120.0	
**Brand**					
Brand A	32	2,255.6 (758.9)	2,165.2 (1,641.9–2,619.4)	1,070.6–4,057.1	<.001
Brand B	11	2,544.5 (595.9)	2,700.0 (2,280–2,909.1)	1,290.9–3,300.0	
Brand C	13	1,777.9 (526.5)	1,733.3 (1,300–2,037.5)	1,100.0–2,933.3	
Others	229	1,769.1 (1,060.3)	1,677.7 (1,153.3–2,050.0)	0–8,000.0	
**Recipe**					
Spicy	120	1,879.5 (879.6)	1,741.7 (1,314.7–2,361.1)	190.0–5,583.3	.68
Nonspicy	165	1,835.5 (1,107.1)	1,800.0 (1,265.2–2,253.5)	0–8,000.0	
**Type of noodles**					
Egg noodles	107	1975.8 (597.0)	1,916.7 (1,521.2–2,385.7)	900.0–3,378.8	<.001
Rice stick noodles	6	2303.3 (1,125.7)	2,255.3 (1,472.7–3,054.5)	800.0–3,981.8	
Rice vermicelli	7	2270.5 (582.3)	2,300.0 (1,665.0–2,909.1)	1,370.4–2,909.1	
Glass noodles	14	2873.3 (1,066.0)	2,896.1 (2,542.9–3,750)	877.8–4,657.1	
Ramen	69	1735.9 (1,086.8)	1,566.7 (1,153.3–1,839.6)	659.6–5,853.7	
Others	34	1632.7 (1,584.5)	1,225.0 (580.2–2,107.7)	190.0–8,000.0	
**Container**					
Bag	182	1,848.8 (1,094.7)	1,665.8 (1,276.6–2,166.7)	0–8,000.0	.10
Cup	103	1,863.3 (864.9)	1,906.3 (1,289.1–2,462.5)	40.0–4,657.1	
**Preparation**					
Soup	207	2,116.8 (1,037.4)	1,913.0 (1,589.9–2,542.9)	0–8,000.0	<.001
Without soup	78	1,156.6 (500.2)	1,076.9 (894.7–1,401.0)	93.8–2,542.9	
**Country of manufacture**					
Thailand	183	1,939.5 (853.4)	1,885.7 (1,458.8–2,519.5)	0–4,657.1	<.001
South Korea	65	1,226.7 (467.8)	1,265.2 (925.0–1,580.0)	190.0–2,436.4	
China	10	1,704.6 (195.4)	1,761.7 (1,530.0–1,880.0)	1,372.7–1,940.0	
Japan	10	3,695.5 (1,735.8)	2,909.0 (2,008.2–5,583.3)	1,913.0–5,853.7	
Others	17	2,337.4 (1,839.8)	1,507.3 (1,386.4–2,257.1)	964.7–8,000.0	

a Calculated by using Mann–Whitney *U* test.

### Trends in sodium content, 2018 to 2021

Median (IQR) sodium content decreased by 11.6%, from 1,980.0 (1,353.8–2,542.9) mg/100 g in 2018 to 1,750.0 mg/100 g in 2021 (*P* = .02). The decline from the 2015 baseline followed a logarithmic trend (*R* ^2^ = 0.99), with the steepest decline from 2015 to 2018 and smaller reductions thereafter (Figure). Overall, sodium reductions were significant among rice porridge/soup (−28.8%, *P* = .004), brand C (−30.2%, *P* = .003), egg noodles (−9.9%, *P* = .03), cup products (−18.2%, *P* = .005), and products manufactured in Thailand (−23.1%, *P* < .001) and China (−9.0%, *P* = .007). In contrast, Japanese products increased in sodium content (+29.3%, *P* = .02).

**Figure:**
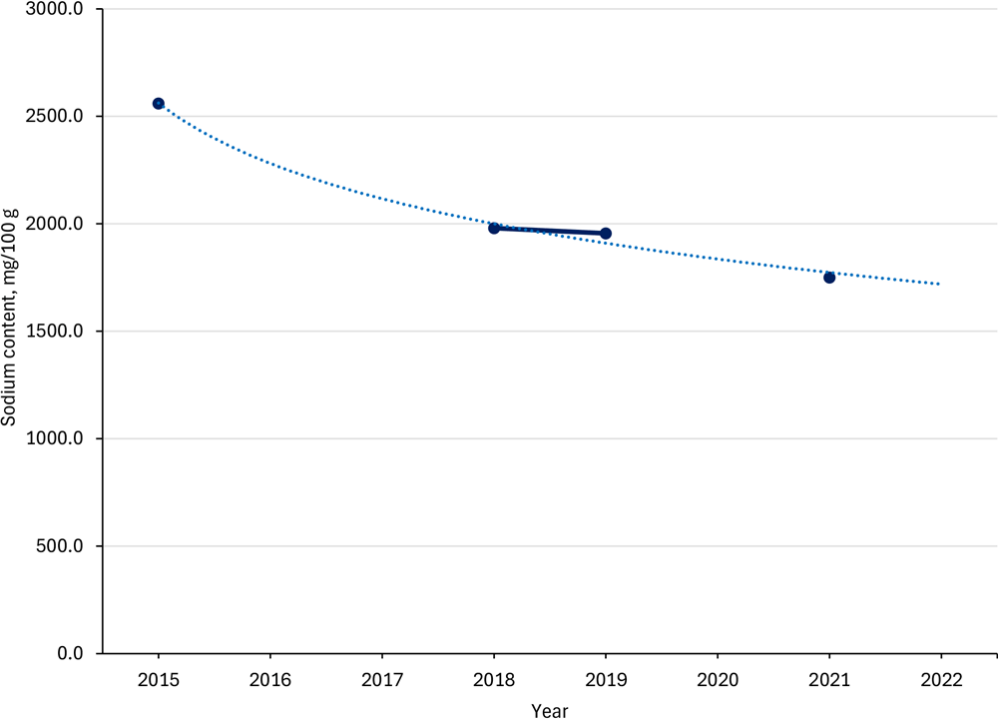
Trend in median sodium content (mg/100 g) of instant foods sold in Thailand, 2015–2021. Circles indicate observed median sodium content. The 2015 value is derived from a previous survey of Thai ready-to-eat packaged foods (10), and the 2018, 2019, and 2021 values are from the present study. The solid line connects the observed values, and the dotted line shows the fitted logarithmic trend (*R²* = 0.99).

**Table:** 

Year	No. of products	Median sodium content, mg/100 g
2015	458	2560.0
2016	—	—
2017	—	—
2018	201	1980.0
2019	302	1955.7
2020	—	—
2021	285	1750.0

### Changes in identical products, 2018 vs 2021

For 57 products available in both 2018 and 2021, the median (IQR) change in sodium content was 0 (−240.0 to 0) mg/100g (*P* = .06) (Table 2). Sodium content decreased in 33.3% of these products, increased in 19.3%, and was unchanged in 47.4%. The median (IQR) reduction was significant among cup products (0 [−525.0 to 0] mg/100 g; *P* =.02) but not among bagged products (0 [−123.9 to 17.5] mg/100 g; *P* = .44). Median (IQR) sodium content decreased significantly in products with soup (0 [−281.3 to 0] mg/100 g; *P* = .004) but increased in products without soup (138.9 [0 to 233.0] mg/100 g; *P* = .03). Among products manufactured in Thailand, median (IQR) sodium content also decreased significantly (0 [−281.3 to 0] mg/100 g; *P* = .04).

**Table 2:** Change in the Sodium Content (mg/100 g) of Identical Instant Food Products From 2018 to 2021, Thailand

Characteristic	No.	Mean (SD)	Median (IQR)	Range	*P* value^a^
**Total**	57	−99.5 (288.4)	0 (−240.0 to 0)	−700.0 to 527.3	.06
**Type**					
Noodles	46	−74.6 (278.4)	0 (−175.0 to 0)	−689.3 to 527.3	.17
Rice porridge/soup	11	−203.5 (319.8)	0 (−657.1 to 0)	−700.0 to 85.7	.13
**Brand**					
Brand A	11	−186.4 (288.0)	0 (−586.0 to 0)	−700.0 to 0	.047
Brand B	9	84.2 (337.0)	33.3 (−72.7 to 366.7)	−450.0 to 527.3	.44
Brand C	2	112.8 (159.5)	112.8 (0.0 to 225.5)	0.0 to 225.5	.32
Others	35	−131.5 (264.7)	0 (−281.3 to 0)	−689.3 to 239.0	.06
**Recipe**					
Spicy	19	21.1 (241.8)	0 (0.0 to 150.0)	−586.0 to 500.0	.30
Nonspicy	38	−159.8 (293.7)	0 (−433.3 to 0)	−700.0 to 527.3	.004
**Type of noodles**					
Egg noodles	34	−84.0 (284.1)	0 (−280.0 to 1.7)	−589.7 to 500.0	.24
Rice stick noodles	1	−689.3^b^	—b	—b	—b
Rice vermicelli	1	527.3^b^	—b	—b	—b
Glass noodles	4	−43.8 (87.5)	0 (−87.5 to 0)	−175.0 to 0	.32
Ramen	5	−48.0 (107.3)	0 (0 to 0)	−240.0 to 0	.32
Others	1	0^b^	—b	—b	—b
**Container**					
Bag	40	−50.3 (270.9)	0 (−123.9 to 17.5)	−700.0 to 527.3	.44
Cup	17	−215.1 (303.3)	0 (−525.0 to 0)	−689.3 to 0	.02
**Preparation**					
Soup	49	−138.4 (288.2)	0 (−281.3 to 0)	−700.0 to 527.3	.004
Without soup	8	138.8 (142.7)	138.9 (0 to 233.0)	0 to 366.7	.03
**Country of manufacture^c^**					
Thailand	43	−110.0 (299.9)	0 (−281.3 to 0)	−700.0 to 527.3	.04
South Korea	8	6.7 (18.4)	0 (0 to 0.9)	0 to 52.2	.16
China	3	−256.7 (265.4)	−240.0 (−530.0 to 0)	−530.0 to 0	.17
Others	3	−74.4 (532.6)	227.1 (−689.3 to 239.0)	−689.3 to 239.0	>.99

a Calculated by using Wilcoxon signed-rank test.

b Because the category comprised only 1 product, measures of dispersion and significance testing were not applicable.

c We found no identical products for Japan.

### Healthier Choice products in 2021

Sixty products (21.1%) met Healthier Choice criteria in 2021. Certification was more common for rice porridge/soup than for noodles (45.8% vs 16.0%, *P* < .001) and for Thai products compared with imported products (30.1% vs 11.8%, *P* < .001).

### Trends in Healthier Choice certification

The proportion of Healthier Choice products increased significantly from 4.5% (9 of 201 products) in 2018 to 21.1% (60 of 285 products) in 2021 (*P* < .001) (Table 3). We observed increases across both noodles and rice porridge/soup, nonspicy recipes, bag and cup packages, and Thai-manufactured products. The largest increases occurred among “other” brands: from 1.3% (2 of 151 products) in 2018 to 18.8% (43 of 229 products) in 2021 (*P* < .001).

**Table 3:** Percentage of Healthier Choice Products,^a^ by Food Characteristics, Thailand, 2018–2021

Characteristic	2018		2019		2021		*P* value
	All products, no.	Healthier Choice products, no. (%)	All products, no.	Healthier Choice products, no. (%)	All products, no.	Healthier Choice products, no. (%)	
**Total**	201	9 (4.5)	302	35 (11.6)	285	60 (21.1)	<.001^b^
**Type**							
Noodles	170	7 (4.1)	258	18 (7.0)	237	38 (16.0)	<.001^b^
Rice porridge/ soup	31	2 (6.5)	44	17 (38.6)	48	22 (45.8)	<.001^b^
**Brand**							
Brand A	26	5 (19.2)	53	12 (22.6)	32	11 (34.4)	.20^b^
Brand B	12	2 (16.7)	18	2 (11.1)	11	2 (18.2)	>.99^c^
Brand C	12	0 (0.0)	21	1 (4.8)	13	4 (30.8)	.10^c^
Others	151	2 (1.3)	210	20 (9.5)	229	43 (18.8)	<.001^b^
**Recipe**							
Spicy	73	6 (8.2)	138	16 (11.6)	120	22 (18.3)	.05^b^
Nonspicy	128	3 (2.3)	164	19 (11.6)	165	38 (23.0)	<.001^b^
**Type of noodles**							
Egg noodles	113	7 (6.2)	171	18 (10.5)	107	32 (29.9)	<.001^b^
Rice stick noodles	2	0	8	0	6	1 (16.7)	>.99^c^
Rice vermicelli	2	0	5	0	7	0	—
Glass noodles	5	0	8	0	14	2 (14.3)	>.99^c^
Ramen	23	0	48	0	69	3 (4.3)	.57^c^
Others	25	0	18	0	34	0	—
**Container**							
Bag	123	3 (2.4)	177	21 (11.9)	182	38 (20.9)	<.001^b^
Cup	78	6 (7.7)	125	14 (11.2)	103	22 (21.4)	.01^b^
**Preparation**							
Soup	166	5 (3.0)	238	23 (9.7)	207	38 (18.4)	<.001^b^
Without soup	35	4 (11.4)	64	12 (18.8)	78	22 (28.2)	.05^b^
**Country of manufacture**							
Thailand	108	9 (8.3)	180	35 (19.4)	183	55 (30.1)	<.001^b^
South Korea	50	0	76	0	65	3 (4.6)	.26^c^
China	17	0	20	0	10	0	—
Japan	17	0	12	0	10	0	—
Others	9	0	14	0	17	2 (11.8)	.53^c^

Abbreviation: —, not applicable.

a The Healthier Choice logo is a voluntary front-of-package symbol, introduced in 2016, certifying foods that meet category-specific nutrient criteria, including sodium limits (13).

b Calculated by using χ^2^ test.

c Calculated by using Fisher exact test.

## Discussion

This study provides new evidence on trends in sodium content of instant foods sold in Thailand from 2018 to 2021. The overall median sodium level declined modestly, and the proportion of Healthier Choice products increased nearly 5-fold. These findings reflect measurable progress in several national policies introduced since 2016, which include the sodium-reduction strategy, mandatory nutritional labeling, and voluntary industry targets. However, despite these efforts, product reformulation remains inconsistent across product categories and brands.

The 2021 median sodium level of 1,750 mg/100 g was more than double the WHO global benchmark for noodles and similar products (18). Products with soup contained nearly twice the sodium of those without soup, consistent with findings from Malaysia and South Korea (19,20). Brands with the largest market share tended to have the highest sodium, indicating the importance of engaging leading manufacturers in reformulation.

The reduction of 230 mg/100 g over 3 years aligns with Thailand’s voluntary target of a 10% reduction every 2 years under the Sodium Reduction Initiative (21). Compared with high-income countries, where pooled decreases averaged 36 mg/100 g (22), Thailand achieved greater reductions. However, we found no significant changes in matched products, indicating that overall reductions were driven by market turnover rather than active reformulation. Specifically, of the 201 products surveyed in 2018, only 57 were also found in 2021; the remaining 144 were therefore considered discontinued, while 228 new products had entered the market. This high turnover suggests the industry prefers launching lower-sodium alternatives — often to attain Healthier Choice certification — over altering the formulations of established core products. Adoption of sales-weighted monitoring, as used in Canada and the UK (23,24), would better reflect consumer exposure.

The proportion of Healthier Choice products increased substantially, but Thailand’s sodium threshold for instant noodles (≤1,000 mg/50 g) remains higher than criteria in neighboring countries like Malaysia and Singapore (25,26). Thus, even certified products may still contribute to excessive sodium intake. Revising these thresholds downward to align with WHO recommendations (800 mg/100 g) (5) is essential to improve their effectiveness. Experiences from Latin America underscore that achieving product-level targets alone may not reduce population intake (27).

To accelerate progress, Thailand’s sodium reduction policies could be strengthened by using WHO’s SHAKE (Surveillance, Harness industry, Adopt standards for labelling and marketing, Knowledge, and Environment) technical package (28). Our findings showed that established products underwent almost no reformulation, indicating that voluntary agreements may be insufficient for inherently high-sodium foods. For these products, setting mandatory maximum sodium levels — as recommended in the “Harness industry” (“H”) component of SHAKE — would likely be more effective. Furthermore, because condiments are the primary source of sodium among people in Thailand, reformulating packaged foods must be combined with environmental interventions and public education to successfully reduce sodium intake in the overall population.

### Limitations

This study is the first to systematically evaluate the sodium content of instant foods in Thailand after policy implementation. However, several limitations should be noted. First, reliance on surveys conducted in the Bangkok Metropolitan Region may not reflect products available in other regions, particularly border areas. Second, sodium levels were derived from nutrition labels rather than laboratory analyses; although regulated by the Thai FDA, discrepancies may occur. Third, differences in survey methodologies between 2018–2019 and 2021 could have introduced variability, although consistent inclusion and exclusion criteria were applied.

### Recommendations

1) The Institute of Nutrition, Mahidol University, should revise sodium limits for instant foods to align with WHO recommendations and Thai dietary guidelines. Gradual lowering would support feasibility for industry compliance.

2) The Bureau of Nutrition and the Division of Noncommunicable Diseases should establish maximum and average sodium targets for instant foods disaggregated by noodles and rice porridge/soup, using 2021 data as the baseline.

3) The Thai FDA should implement systematic monitoring of the sodium content of instant foods, including annual retail surveys, mandatory reporting of nutritional analysis of reformulated or newly launched products, and transparent public reporting of industry progress.

4) The Low Salt Network and its partners should communicate findings by food characteristics to raise public awareness and empower consumers — particularly those with hypertension and chronic kidney disease — to choose lower-sodium products and sustain pressure on the food industry to reformulate.

### Conclusion

Sodium levels in instant foods sold in Thailand declined modestly from 2018 to 2021, and the availability of Healthier Choice products increased substantially. However, most items still exceed WHO benchmarks, and reformulation is inconsistent. Stronger measures — such as stricter Healthier Choice thresholds, mandatory sodium targets, and systematic monitoring — are needed to accelerate sodium reduction and support national efforts to lower the prevalence of hypertension and cardiovascular disease.
